# A new species of *Giardia* Künstler, 1882 (Sarcomastigophora: Hexamitidae) in hamsters

**DOI:** 10.1186/s13071-018-2786-8

**Published:** 2018-03-20

**Authors:** Zhangxia Lyu, Jingru Shao, Min Xue, Qingqing Ye, Bing Chen, Yan Qin, Jianfan Wen

**Affiliations:** 1 State Key Laboratory of Genetic Resources and Evolution, Kunming Institute of Zoology, Chinese Academy of Sciences, Kunming, Yunnan 650223 China; 2 Kunming College of Life Science, University of Chinese Academy of Sciences, Kunming, Yunnan 650204 China; 3 Kunming Institute of Botany, Chinese Academy of Sciences, Kunming, Yunnan 650201 China

**Keywords:** *Giardia cricetidarum* n. sp., Host range and specificity, Morphology, Phylogenetic analysis

## Abstract

**Background:**

*Giardia* spp. are flagellated protozoan parasites that infect humans and many other vertebrates worldwide. Currently seven species of *Giardia* are considered valid.

**Results:**

Here, we report a new species, *Giardia cricetidarum* n. sp. in hamsters. Trophozoites of *G. cricetidarum* n. sp. are pear-shaped with four pairs of flagella and measure on average 14 μm (range 12–18 μm) in length and 10 μm (range 8–12 μm) in width. The trophozoites of the new species are generally larger and stouter than those of most of the other *Giardia* spp. and exhibit the lowest length/width ratio (*c.*1.40) of all recognized *Giardia* species. Cysts of *G. cricetidarum* n. sp. are ovoid and measure on average 11 μm (range 9–12 μm) in length and 10 μm (range 8–10 μm) in width and are indistinguishable from the cysts of other *Giardia* species. Molecular phylogenetic analyses based on *beta-giardin*, *small subunit rRNA*, and *elongation factor-1 alpha* loci all demonstrated that *G. cricetidarum* n. sp. is genetically distinct from all currently accepted *Giardia* spp. Investigation of the host range indicated that the new species was only found in hamsters (including *Phodopus sungorus*, *P. campbelli* and *Mesocricetus auratus*), while all the other described mammal-parasitizing species (*G. muris*, *G. microti* and *G. intestinalis*) each infect multiple hosts. Cross-transmission studies further demonstrated the apparent host specificity of *G. cricetidarum* n. sp. as it only infected hamsters. Trophozoites were found in high numbers in hamster intestines (5 × 10^5^ – 5 × 10^6^) and was rarely detected co-infecting with other *Giardia* spp. in the common hamster, suggesting it has some advantages in parasitizing hamsters.

**Conclusions:**

This study has identified a new species of *Giardia*, which appears to be specific to hamsters, and together with the three other mammal-parasitizing *Giardia* species with different host ranges, may be able to be used as a model system for the study of evolutionary divergence of host parasitism strategies in *Giardia*.

**Electronic supplementary material:**

The online version of this article (10.1186/s13071-018-2786-8) contains supplementary material, which is available to authorized users.

## Background

Since Antony van Leeuwenhoek first observed and recorded Diplomonadida in 1681, the flagellated protozoan intestinal parasite, *Giardia* Künstler, 1882, has attracted the attention of scientists for many reasons in the past 300 years [1]. *Giardia* spp. are the most common intestinal protozoan parasites of humans and many other vertebrates worldwide; infection can cause giardiasis, the symptoms of which include acute or chronic diarrhea, nausea and weight loss. Previously up to 51 species of *Giardia* were incorrectly described on the basis of assumed host specificity [2]. Currently seven species of *Giardia* are considered valid: *G. agilis* Künstler, 1882 in amphibians, *G. ardeae* Filice, 1952 and *G. psittaci* Filice, 1952 in birds, *G. microti* Filice, 1952 and *G. muris* Filice, 1952 in rodents, *G. intestinalis* Lambl, 1859 (syns. *G. duodenalis* and *G. lamblia*) in most vertebrates including human beings [3], and the most recently described species, *G. peramelis* Hillman, 2016, in Australian bandicoots (*Isoodon obesulus* Driessen) [4].

The taxonomy of *Giardia* spp. is usually based on morphological characters of the trophozoites and cysts [3]. The shape and size of the trophozoites and cysts are the most common characters used for describing and identifying *Giardia* spp. Today, sequence analysis at multiple loci is essential for the identification and classification of *Giardia*, and the following genes are most widely used: the *glutamate dehydrogenase*, *beta-giardin*, *elongation factor-1 alpha*, *triose phosphate isomerase* and *small subunit rRNA* (*SSU* rRNA) [5]. Within *G. intestinalis*, genetic analysis has identified eight assemblages, A-H: assemblages A and B in humans and other mammals; assemblages C and D in dogs and other canids; assemblage E mainly in ungulates, assemblage F mainly in cats; assemblage G in rats and mice; and assemblage H in marine mammals [6, 7].

In the present study, morphological and molecular characterisation and experimental cross-transmission studies were used to describe a new species, *G. cricetidarum* n. sp. in hamsters.

## Methods

### Collection and purification of trophozoites and cysts

*Giardia* trophozoites were collected from hamsters (*Phodopus sungorus*, Kunming, China) which were bought from pet markets. The hamsters were anesthetized to death with chloroform and then their intestines were removed and cut into 0.5 cm segments. Segments of intestines were collected in 2 ml centrifuge tubes with normal saline. The centrifuge tubes were chilled in ice for 20 min. The suspension was briefly centrifuged at 1,000× *g* for 1 s to remove the large fragments. The supernatant was removed into new centrifuge tubes and then was centrifuged at 500× *g* for 5 min to concentrate the trophozoites. The pellet was resuspended with TYI-S-33 medium and cultivated at 37 °C for 3 h [8]. Then the medium was replaced with fresh medium when the majority of *G. cricetidarum* cells had adhered to the wall of the centrifuge tube [9]. The medium was replaced with phosphate buffered saline (PBS) buffer and the trophozoites were cultivated at 37 °C for 2 h. The centrifuge tubes were chilled in ice for 20 min and centrifuged at 10,000× *g* for 2 min, and the supernatant was removed to collect the trophozoites, which were frozen at -20 °C for later use. Cysts were obtained from the feces of infected hamsters and prepared for morphometric and host range studies by re-suspending in water and filtering the suspension through a 60 mesh sieve and centrifugation at 1500× *g* for 10 min. A 33% zinc sulfate solution with the same amount of sediment was added to resuspend the sediment, and centrifuged at 1200× *g* for 15 min. The supernatant was diluted with 4 volumes of water and centrifuged again at 1500× *g* for 10 min. Then the last two steps were repeated twice to further purify the cysts.

### Host range and experimental cross-transmission studies

The host range was examined by screening feces and intestinal contents from a range of vertebrate animals including frogs (wild), parrots (domestic), mice (domestic and wild), small oriental voles (wild) and hamsters (domestic), from different areas or markets, with at least 10 individuals examined for each species. These animals were anesthetized to death with chloroform and their intestines were removed and cut into 0.5 cm segments. The segments of intestines were collected in 2 ml centrifuge tubes with normal saline and were chilled in ice for 20 min. The suspension was briefly centrifuged at 1000× *g* for 1 s to remove the large fragments. The supernatant was removed into new centrifuge tubes and centrifuged at 500× *g* for 5 min to concentrate *Giardia*. The sediment was resuspended with normal saline and then examined under a bright field microscope. Cross-transmission studies were performed by adding ~10^6^ purified cysts of *G. cricetidarum* n. sp. to 80 ml of the drinking water for *Rattus norvegicus*, *Mus musculus* and *Oryctolagus cuniculus* f. *domesticus* (*n* = 10 for each species), after first confirming that their feces was negative for *Giardia* spp. by microscopy. After two weeks, the animals were examined for infection by visual identification of intestines and feces according to Farthing et al. [10]. Ten *Giardia*-uninfected hamsters were also infected in the same way and used as controls. All the animals were housed in different cages.

### Microscopy

For haematoxylin and eosin staining, PBS solution containing *G. cricetidarum* trophozoites was dried on a glass slide; the glass slide was placed into Carnoy's fluid for 15 min and washed with PBS buffer for 5 min. Then the glass slide was processed through the following steps: stained with haematoxylin for 30 min and then rinsed with tap water; dipped in hydrochloric-alcohol solution (saturated hydrochloric acid diluted in 70% alcohol, with a final concentration of 1% hydrochloric acid) for 7 s and then rinsed with tap water; dipped in 2% ammonium hydroxide for 10 s and then rinsed with tap water; stained with 0.5% eosin (acetic acid in eosin solution with a final concentration of 2% acetic acid) for 10 min and washed by water; dehydrated through an ascending ethanol series (70%, 80%, 90%, 95% and 100%); cleared in dimethylbenzene and mounted in neutral balsam.

Slides of trophozoites and cysts were prepared as described above. All slides were examined under oil immersion by a 100× HCX PL APO objective on a Leica DM2500 microscope (Leica, Wetzlar, Germany). The images were captured by a Leica DFC450 C digital camera. The length and width of the trophozoites (*n* = 20 per sample) and cysts (*n* = 10 per sample) from 87 samples were measured. Sample handling and photomicrographs of scanning electron microscope were processed at the Kunming Medical University (Kunming, China) using HITACHI 3700N (Tokyo, Japan).

### DNA extraction, PCR amplification and sequencing

Genomic DNA of the new species was extracted from purified trophozoites using the Qiagen QIAamp DNA Mini Kit (Qiagen, Hilden, Germany) according to the manufacturer's protocol. Total fecal DNA was extracted from the host animals using the TIANamp Stool DNA Kit (Tiangen, Beijing, China). The primers specific to both ends of the sequences of the three genes, *SSU* rRNA, *β-giardin* and *elongation factor-1 alpha*, were designed according to the conserved sequences of these genes of *G. intestinalis* (50586 isolate) using PRIMER PREMIER program version 5.00 (Biosoft International) [11], and the expected product lengths of the *SSU* rRNA, *β-giardin* and *elongation actor-1 alpha* were 950 bp, 500 bp and 630 bp, respectively (see primer sequences in Additional file 1). The primers were used to amplify the sequences of the three genes from all *Giardia* samples by PCR. The PCR reactions were set up in 25 μl containing 1× GC buffer, 0.08 mM dNTP, 1 μM of each primer, 0.25 μl (0.5U) LA-Taq DNA polymerase (Takara, Tokyo, Japan) and 0.5–1 μl of cell sample or DNA. Thermocycling conditions were as follows: 94 °C for 2 min followed by 32 cycles of 94 °C for 30 s, 52 °C for 30 s and 72 °C for 30 s, followed by 72 °C for 10 min.

The PCR products were purified using the Wizard SV Gel and PCR clean-up system kit (Qiagen), and cloned into pMD-19T vectors using TaKaRa pMD-19T VectorCloning Kit (TaKaRa, Tokyo, Japan). The ligation products were transformed into DH5α chemically competent *E. coli*. Colony PCR was carried out with the vector-specific primers provided in the kit, and colonies were selected and Sanger-sequenced using vector-specific forward and reverse primers by Sangon Biotech (Shanghai, China).

### Molecular phylogenetic analysis

In order to perform the phylogentic analysis, we sequenced, identified and retrieved the *elongation factor-1 alpha* of *G. cricetidarum* n. sp. (GenBank: MG733773), *G. intestinalis* strain ATCC 50803 (GenBank: KX131163), *G. intestinalis* strain Ad-23 (GenBank: AF069572), *G. intestinalis* strain P-15 (GenBank: AF069571), *G. psittaci* (GenBank: AB714979), *G. ardeae* (GenBank: AF069567), *G. muris* (GenBank: AF069566), *Spironucleus vortens* (GenBank: U94406); *SSU * rRNA of *G. cricetidarum* n. sp. (GenBank: MF185957), *G. muris* (GenBank: MF185956), *G. microti* (GenBank: MF185958), *Spironucleus* sp. (GenBank: FM897198), *S. barkhanu* (GenBank: DQ186590), *G. psittaci* (GenBank: AF473853.1), *G. muris* (GenBank: X65063 S53320), *G. ardeae* (GenBank: Z17210 S53313), *G. microti* (GenBank: AF006677), *G. intestinalis*(dog) (GenBank: AF199449), *G. intestinalis* Assemblage A isolate WB GL50803 r0019 (GenBank: M54878 M19); and *β-giardin* sequences of *G. cricetidarum* n. sp. (GenBank: MF185953), *G. agilis* (GenBank: MF185954), *G. microti* (GenBank:MF185955), *G. intestinalis* Assemblage_A (GenBank: KJ363393), *G. intestinalis* Assemblage_B (GenBank: KJ363389), *G. intestinalis* Assemblage_D (GenBank: KJ027418), *G. intestinalis* Assemblage_E (GenBank: KJ363399), *G. intestinalis* Assemblage_F (GenBank: KJ027424), *G. muris* (GenBank: EF455599), *G. muris* (GenBank: AY258618), *G. psittaci* (GenBank: AB714977).

The number of isolates of *G. cricetidarum* n. sp. for each locus sequenced is as follows: 8 for *SSU* rRNA; 12 for *β*-*giardin*; and 9 for *elongation factor-1 alpha*. The other sequences used in this work were all retrieved from the GenBank database (see accession numbers in Additional file 2: Table S1).

The maximum likelihood phylogenetic trees based on *SSU* rRNA, *β-giardin*, and *elongation factor-1 alpha* DNA sequences were reconstructed by using the PhyML software [12]. The sequence data of the three loci are from 7, 12 and 7 *Giardia* species or isolates; and two *SSU* rRNA sequences for *Spironucleus* spp. and one *elongation factor-1 alpha* sequence for *Spironucleus vortens* were used as outgroups. Multiple sequence alignments were performed with ClustalW 2.0 program [13], and the alignments were visually inspected to eliminate poorly aligned positions. The best-fit DNA model used for reconstructing the maximum likelihood phylogeny was selected by the JModelTest software [14, 15]. Trees were constructed using the PhyML 3.0 based on 9 taxa with 437 nt positions for *SSU* rRNA gene, 12 taxa with 284 nt positions for *β-giardin* gene, and 8 taxa with 550 nt positions for *elongation factor-1 alpha* gene. Tree reliability was determined by using bootstrap analyses with 1000 replicates.

## Results

**Order Diplomonadida Wenyon, 1926**

**Family Hexamitidae Kent, 1881**

**Genus** ***Giardia*** **Künstler, 1882**

***Giardia cricetidarum*** **n. sp.**

***Type-host*:** *Phodopus sungorus* Pallas (Rodentia: Cricetidae), Dzhunfarian hamster.

***Other natural hosts*:** *Phodopus campbelli* and *Mesocricetus auratus*

***Type-locality*:** Kunming, Yunnan, China.

***Type-specimens*:** Frozen *G. cricetidarum* trophozoites were deposited at the Kunming Institute of Zoology (KIZ), Chinese Academy of Sciences, Kunming, Yunnan, China. Hapantotype permanent mounts and photomicrographs were deposited in the Kunming Natural History Museum of Zoology and Kunming Institute of Zoology under accession numbers KIZ170814 and KIZ170925.

***Site of infection*:** Intestine.

***Prepatent period:*** 7–14 days.

***Patent period*:** Unknown.

***Representative DNA sequences*:** Partial sequences of *SSU* rRNA, *beta-giardin* and *elongation factor-1 alpha* genes were submitted to the GenBank database under the accession numbers MF185957, MF185953 and MG733773, respectively.

***ZooBank registration*:** To comply with the regulations set out in article 8.5 of the amended 2012 version of the *International Code of Zoological Nomenclature* (ICZN) [16], details of the new species have been submitted to ZooBank. The Life Science Identifier (LSID) of the article is urn:lsid: zoobank.org:pub:F2CBD145-DE59-4C68-A333-8DE022F86892. The LSID for the new name *Giardia cricetidarum* is urn:lsid:zoobank.org:act:C3001577-97B2-4E05-A277-302A39CC6EAC.

***Etymology:*** The new species is named the Cricetinae Fischer (hamsters), a subfamily of the Cricetidae Fischer.

### Description

**Fig. 1:**
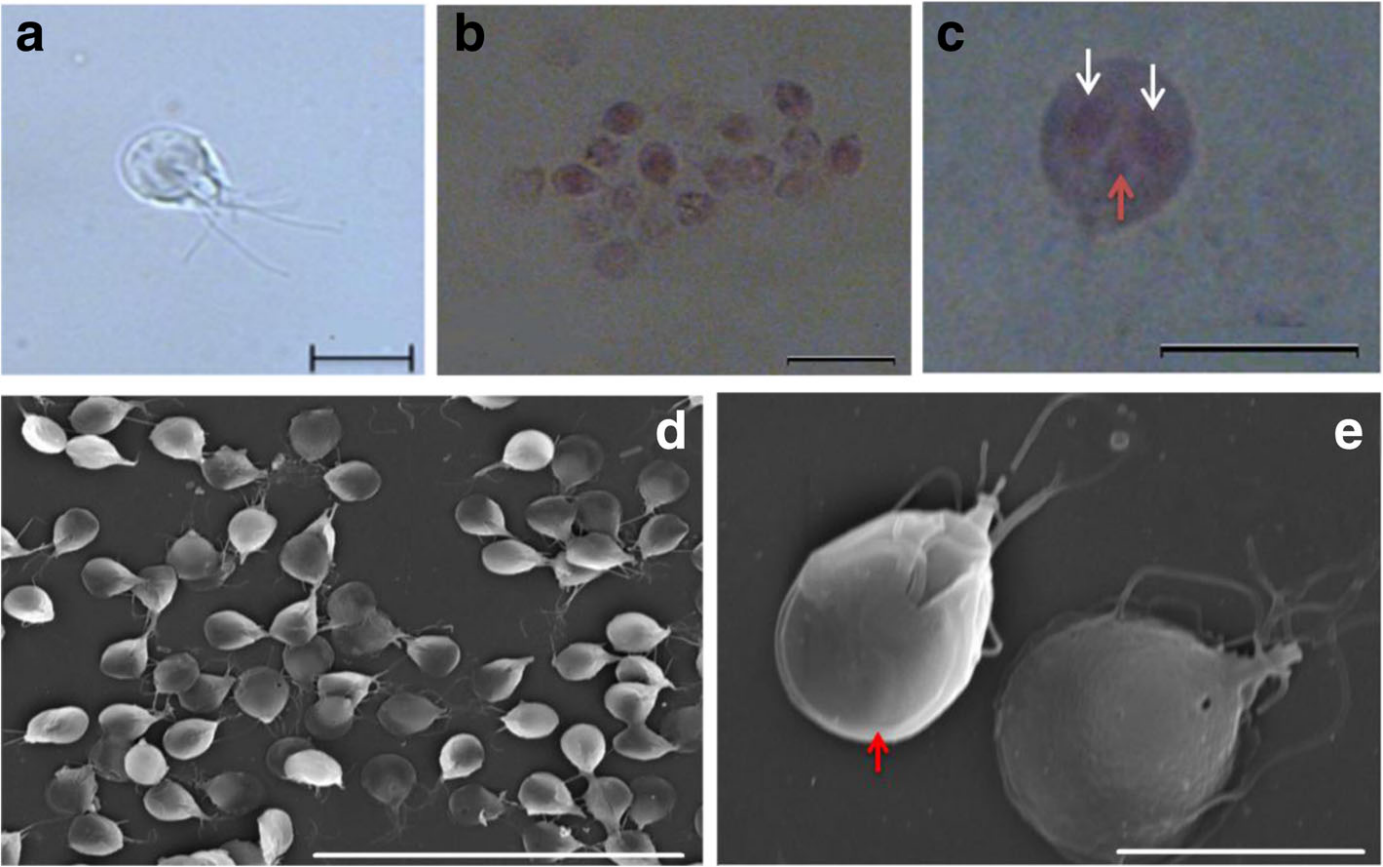
Trophozoites of *Giardia cricetidarum* n. sp. **a** Photomicrographs under bright-field microscope. **b** Stained trophozoites. **c** A stained trophozoite of *G. cricetidarum* (white arrows point to the nucleus and red arrow points to the median body). **d** Trophozoites under scanning electron microscope. **e** Trophozoites under scanning electron microscope, at high magnification (red arrow points the ventral disk). *Scale-bars*: **a**, **c**, **e**, 10 μm; **b**, 20 μm; **d**, 70 μm

***Trophozoite.*** [Based on scanning electron microscope micrographs from 36 samples; Fig. 1d and e.] Trophozoites stout, pyriform, 12–18 (14 ± 1.37) μm long (*n* = 720), 8–12 (10 ± 1.02) μm wide (*n* = 720). Ventral disc 7–8 (8 ± 0.53) μm long (*n* = 50), 6–8 (8 ± 0.67) μm wide (*n* = 50). Two nuclei, 2–3 (3) μm long (*n* = 10), 1–3 (2) μm wide (*n* = 10). Flagella 4 pairs, equal in length, 12–16 (15) μm give range for flagellum length.

**Fig. 2:**
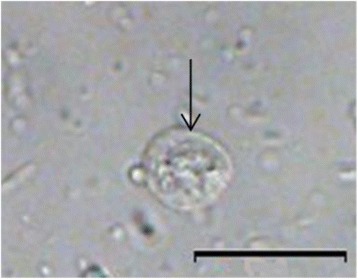
Cyst of *Giardia cricetidarum* (arrowed). *Scale-bar*: 20 μm

***Cyst*.** [Based on photomicrographs of bright field microscope from 36 samples; Fig. 2.] Cysts ovoidal, 9–12 (11 ± 0.96) μm long (*n* = 360), 8–10 (10 ± 0.60) μm wide (*n* = 360).

### Remarks

*Giardia cricetidarum* n. sp. is most similar to *G. muris* on the basis of the morphology of the trophozoites shape and molecular data. The new species differs from the other *Giardia* spp. in: (i) its trophozoites are generally larger and stouter than most of the other *Giardia* species (Fig. 1) and exhibit the lowest length/width ratio (~1.40) among all the described *Giardia* species (Table 1); (ii) it is usually detected in high numbers with high density and all individuals were infected in one infected population; (iii) it was only found in hamsters yet. However, the cysts of *G. cricetidarum* were ovoidal and measured 9–12 (11 ± 0.96) μm long and 8–10 (10 ± 0.60) μm wide (Fig. 2), which are similar to other *Giardia* spp. such as *G. intestinalis*, which are also ovoidal and measure 8–12 μm in length and 7–10 μm in width [17]. Thus, the cysts of *G. cricetidarum* are morphologically indistinguishable from those of other *Giardia* species.

**Table 1:** Trophozoite size for *Giardia cricetidarum* and other *Giardia* spp

Species	Length (μm)	Width (μm)	Length/width ratio
*G. cricetidarum* n. sp.	12–18	8–12	1.40
*G. intestinalis*	12–15	6–8	1.79
*G. muris*	9–12	5–7	1.75
*G. microti*	12–15	6–8	1.79
*G. agilis*	20–30	4–5	5.56
*G. ardeae*	*c.*10	*c.*6.5	1.54
*G. psittaci*	*c.*14	*c.*6	2.33

*Note*: The data for *Giardia* spp. are from Lujan et al. [3]

### The host range of *G. cricetidarum*

During our investigation of the host range of *G. cricetidarum*, we detected *G. intestinalis* in *Rattus norvegicus* (Berkenhout) and *Phodopus sungorus* (Pallas), *G. microti* in *Eothenomys melanogaster* (Milne-Edwards), *G. muris* in *R. norvegicus*, *P. sungorus*, and *G. agilis* in *Babina pleuraden* (Boulenger), but *G. cricetidarum* n. sp. was only found in hamsters, i.e. *P. sungorus*, *P. campbelli* (Thomas) and *Mesocricetus auratus* (Waterhouse). The detailed results are shown in Table 2. Our cross-transmission studies showed that the rodents including *R. norvegicus* and *M. musculus* and lagomorphs including *Oryctolagus cuniculus* f. *domesticus* did not produce detectable infections with *G. cricetidarum* n. sp. while all of the control hamsters were easily infected. This suggests that *G. cricetidarum* n. sp. may specifically parasitize hamsters and has the narrowest host range within all the known mammal-parasitizing *Giardia* spp. However, further research is required to confirm this. No significant loss in weight and no intestinal lesions of hamsters infected with *G. cricetidarum* n. sp. were found.

**Table 2:** *Giardia* spp. identified in the host range investigation of *G. cricetidarum* n. sp

Host species	No. examined	*Giardia* species	Prevalence (%) *n* infected/*n* examined
*Babina pleuraden*	62	*G. agilis*	100 (62/62)
*Rana chaochiaoensis*	34	*G. agilis*	65 (22/34)
*Rattus norvegicus*	23	*G. muris*	17 (4/23)
		*G. intestinalis*	4 (1/23)
*Eothenomys melanogaster*	7	*G. microti*	100 (7/7)
*Phodopus sungorus*	87	*G. cricetidarum* n. sp.	41 (36/87)
		*G. intestinalis*	7 (6/87)
		*G. muris*	3 (3/87)
*Phodopus campbelli*	9	*G. cricetidarum* n. sp.	100 (9/9)
*Mesocricetus auratus*	11	*G. cricetidarum* n. sp.	100 (11/11)

### Comparison of the prevalence features between *G. cricetidarum* and the other mammal-parasitizing *Giardia*

Our investigation revealed an interesting distribution feature of *G. cricetidarum* n. sp. in feeding populations of hamsters (bought from pet markets); for a certain feeding population, either all of the individuals or none of them were infected by *G. cricetidarum*, exhibiting an “all or none” infection pattern. Two of the ten hamster populations examined were infected, thus the infection rate per population was 20% [overall prevalence of 52% (56/107)]. Interestingly the numbers of *G. cricetidarum* trophozoites in all infected hamsters examined were high (5 × 10^5^–5 × 10^6^). This “all or none” prevalence has seldom been documented for the three other mammal-parasitizing *Giardia* species in their corresponding hosts [3]. In the present study, some hamsters co-infected with *G. intestinalis* and *G. muris* were identified but the infection rates of *G. intestinalis* and *G. muris* in all of the hamsters examined were only 6% (6 out of 107) and 3% (3 out of 107), respectively. In addition, hamsters infected with *G. intestinalis* and *G. muris* shed much lower numbers of trophozoites (less than 10^4^) and of the 36 *G. cricetidarum*-infected hamsters, only one and two individuals were co-infected with *G. intestinalis* and *G. muris*, respectively. This suggests that hamsters are the most suitable hosts for *G. cricetidarum* n. sp. rather than other mammals.

### Genetic distinction between *G. cricetidarum* n. sp. and other known *Giardia* species

Genetic distances between *Giardia* species at the three loci (Tables 3, 4 and 5) indicate that the genetic distances between *G. cricetidarum* n. sp. and all other *Giardia* species are equal or greater than the differences between currently accepted *Giardia* species. Although *G. cricetidarum* n. sp. is most genetically close to *G. muris*, it has a significant genetic distance with all of the other *Giardia* spp., including those that can parasitize the same host hamster.

**Table 3:** Genetic distances between *Giardia* spp. for the *SSU* rRNA locus

	Species	1	2	3	4	5	6
1	*G. intestinalis*						
2	*G. psittaci*	0.546					
3	*G. ardeae*	0.107	0.575				
4	*G. microti*	0.017	0.556	0.117			
5	*G. muris*	0.183	0.559	0.158	0.196		
6	*G. peramelis*	0.581	0.592	0.582	0.574	0.603	
7	*G. cricetidarum* n. sp.	0.197	0.575	0.152	0.210	0.098	0.595

**Table 4:** Genetic distances between *Giardia* spp. for the *β-giardin* locus

	Species	1	2	3	4	5
1	*G. intestinalis*					
2	*G. psittaci*	0.139				
3	*G. agilis*	0.087	0.128			
4	*G. microti*	0.100	0.129	0.012		
5	*G. muris*	0.108	0.170	0.099	0.113	
6	*G. cricetidarum* n. sp.	0.104	0.152	0.105	0.113	0.033

**Table 5:** Genetic distances between *Giardia* spp. for the *elongation factor-1 alpha* locus

	Species	1	2	3	4
1	*G. intestinalis*				
2	*G. ardeae*	0.089			
3	*G. muris*	0.121	0.086		
4	*G. psittaci*	0.110	0.124	0.151	
5	*G. cricetidarum* n. sp.	0.111	0.076	0.072	0.137

**Fig. 3:**
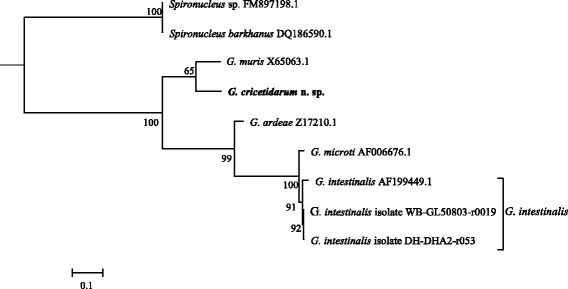
Phylogenetic tree from ML based on *SSU* rRNA sequences indicating that *Giardia cricetidarum* n. sp. is distinct from all *Giardia* spp. currently considered valid. *Spironucleus* spp. were used as the outgroup

**Fig. 4:**
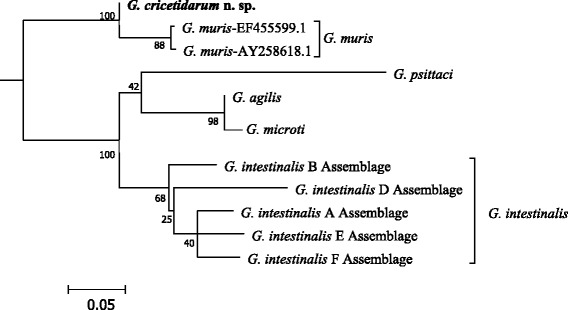
Phylogenetic tree from ML based on *β-giardin* sequences indicating that *Giardia cricetidarum* n. sp. is distinct from all *Giardia* spp. currently considered valid

**Fig. 5:**
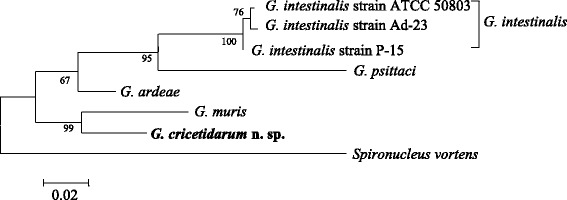
Phylogenetic tree from ML based on *elongation factor-1 alpha* sequences indicating that *G. cricetidarum* n. sp. is distinct from all *Giardia* spp. currently considered valid. *Spironucleus vortens* was used as the outgroup

Phylogenetic analysis of a fragment of the *SSU* rRNA (Fig. 3), the *β-giardin* (Fig. 4) and the *elongation factor-1 alpha* (Fig. 5) loci showed that although *G. cricetidarum* is genetically most closely related to *G. muris*, it is genetically distinct from all other *Giardia* species, including those that can parasitize hamsters.

## Discussion

In the present study, a new species of *Giardia*, *G. cricetidarum* n. sp. was described from hamsters. Cysts of *G. cricetidarum* are difficult to distinguish morphologically from the other described *Giardia* species, but the trophozoites of the new species are distinctly larger and stouter than those in all the other described *Giardia* spp., including the three mammal-parasitizing *Giardia*, i.e. *G. muris*, *G. microti* and *G. intestinalis*. The phylogenetic analyses based on partial sequences of the three loci (*SSU* rRNA, *β-giardin*, and *elongation factor-1 alpha*) all support the genetic distinctness of *G. cricetidarum* n. sp. Our investigations of the host range and cross-transmission studies both suggest that *G. cricetidarum* is a hamster-specific parasite; however further evidence is required to confirm this. Therefore, the combination of evidence based on biological and molecular data strongly supports that *G. cricetidarum* n. sp. is not only a new species of *Giardia*, but also a mammal-parasitizing *Giardia* with the narrowest host range found so far. Furthermore, we found several special prevalence features of this new species: (i) “all or none” prevalence pattern in hamster populations; (ii) heavy infections in the intestines of infected hamsters without observed clinical signs; and (iii) rare co-infections with other *Giardia* spp. in the common hamster. These features imply that *G. cricetidarum* n. sp. obviously has some advantages in parasitizing hamsters over other mammal-parasitizing *Giardia*. Therefore, the new species, together with the three other mammal-parasitizing *Giardia* spp. with wider host ranges, may be able to be used as a model system for the study of evolutionary differences in the host parasitism strategy of *Giardia* species.

## Conclusion

A new species of *Giardia*, *G. cricetidarum* n. sp. is described. The identification of *G. cricetidarum* may not just add a new member to the ancient genus *Giardia*, but may benefit studies on parasitic adaptation and evolutionary differences in the host parasitism strategies of *Giardia* spp.

## Additional files

Additional file 1: The primer pairs designed for amplification of *SSU* rRNA, *β-giardian* and *elongation factor-1 alpha. (PDF 16 kb)*

Additional file 2: **Table S1.** The *SSU* rRNA, *β-giardian* and *elongation factor-1 alpha* sequences retrieved from GenBank and used for molecular phylogenetic analyses. (PDF 71 kb)

## Abbreviations

SSU rRNA: small subunit ribosomal RNA
PBS: phosphate buffered saline
PCR: polymerase chain reaction
