# Supplementary material for: A new species of Giardia Künstler, 1882 (Sarcomastigophora: Hexamitidae) in hamsters
Source: Parasit Vectors. 2018 Mar 20;11:202. doi: 10.1186/s13071-018-2786-8 (PMC5861651; doi:10.1186/s13071-018-2786-8)
Supplement: Supplementary file 1 — The primer pairs designed for amplification of SSU rRNA, β-giardian and elongation factor-1 alpha. (PDF 16 kb) [file 13071_2018_2786_MOESM1_ESM.pdf]

The primer pairs designed for the PCR experiments

For SSU rRNA:

(Sense primer) 5' AGCAGCCGCGGTAATTCC 3'

3' (680) TCGTCGGCGCCATTAAGG (697) 5'

(Anti-sense primer) 5' CCTTGTTACGACTTCTCCTTCC 3'

3' (1629) GGAACAATGCTGAAGAGGAAGG (1608) 5'

For β-giardian:

(Sense primer) 5' GCGAGGAGGTCAAGAAGTC 3'

3' (11) CGCTCCTCCAGTTCTTCAG (29) 5'

(Anti-sense primer) 5' GAGCGTGTTGACGATCTTGT 3'

3' (510) CTCGCACAACTGCTAGAACA (491) 5'

For elongation factor-1 alpha:

(Sense primer) 5' TCGACCAGCTCAAGGACGA 3'

3' (3) AGCTGGTCGAGTTCCTGCT (21) 5'

(Anti-sense primer) 5' CGAAGACGACCTTCATCCC 3'

3' (632) GCTTCTGCTGGAAGTAGGG (614) 5'
