# Supplementary material for: A new species of Giardia Künstler, 1882 (Sarcomastigophora: Hexamitidae) in hamsters
Source: Parasit Vectors. 2018 Mar 20;11:202. doi: 10.1186/s13071-018-2786-8 (PMC5861651; doi:10.1186/s13071-018-2786-8)
Supplement: Supplementary file 2 — Table S1. The SSU rRNA, β-giardian and elongation factor-1 alpha sequences retrieved from GenBank and used for molecular phylogenetic analyses. (PDF 71 kb) [file 13071_2018_2786_MOESM2_ESM.pdf]

Giardia β-*giardin* genes from GenBank

Assemblage or isolate	accession nember
G. intestinalis Assemblage_A	KJ363393
G. intestinalis Assemblage_B	KJ363389
G. intestinalis Assemblage_D	KJ027418
G. intestinalis Assemblage_E	KJ363399
G. intestinalis Assemblage_F	KJ027424
G. muris	EF455599
G. muris	AY258618
G. psittaci	AB714977

Giardia SSU rRNA genes from GenBank

Assemblage or isolate	accession nember
Spironucleus sp.	FM897198
S. barkhanu	DQ186590
G. psittaci	AF473853.1
G. muris	X65063 S53320
G. ardeae	Z17210 S53313
G. microti	AF006677
G. intestinalis(dog)	AF199449
G. intestinalis Assemblage A isolate WB GL50803 r0019	M54878 M19500

G. intestinalis Assemblage A2 isolate DH DHA2 r053	U09492

Giardia EF1A genes from GenBank

Assemblage or isolate	accession nember
Spironucleus vortens	U94406
G. intestinalis strain ATCC 50803	KX131163
G. intestinalis strain Ad-23	AF069572
G. intestinalis strain P-15	AF069571
G. psittaci	AB714979
G. ardeae	AF069567
G. muris	AF069566
